# Prediction of NR3C1 as a methylation marker for the prevention and treatment of gastric cancer in Rhizoma Atractylodis Macrocephalae based on machine learning algorithm and bioinformatics analysis

**DOI:** 10.3389/fgene.2025.1584986

**Published:** 2025-09-09

**Authors:** Shicong Huang, Qian Gu, Yan Chen, Xiaofang Huang, Yuhua Du, Ziying Zhou, Yi Nan, Ling Yuan

**Affiliations:** ^1^ Pharmacy College of Ningxia Medical University, Yinchuan, Ningxia, China; ^2^ Health Administration and Social Services College, Ningxia Polytechnic, Yinchuan, Ningxia, China; ^3^ Ningxia Chinese Medicine Research Center, Yinchuan, Ningxia, China; ^4^ Ningxia Medical University General Hospital, Yinchuan, Ningxia, China; ^5^ Key Laboratory of Ningxia Ethnomedicine Modernization, Ministry of Education, Ningxia Medical University, Yinchuan, Ningxia, China

**Keywords:** Rhizoma Atractylodis Macrocephalae, gastric cancer, methylation, NR3C1, WGCNA, machine learning

## Abstract

**Objective:**

The aim of this study was to identify the markers of Rhizoma Atractylodis Macrocephalae (RAM) for the prevention and treatment of gastric cancer using bioinformatics analysis.

**Methods:**

The main active components of RAM were screened using the Traditional Chinese Medicine Systematic Pharmacology Profiling Platform (TCMSP) and SwissADME, the target genes of RAM were screened using WGCNA and three machine learning algorithms, and the target genes were analyzed clinically and by methylation.

**Results:**

Three core genes, namely, CA2, HSP90AA1, and NR3C1, were screened by WGCNA and three machine learning algorithms. Clinical correlation analysis and epigenetic analyses showed that these genes play the most important role in gastric cancer. In gastric cancer, there was a strong correlation between NR3C1 methylation and its mRNA expression, suggesting that methylation of NR3C1 may be involved in the regulation of its expression. Therefore, methylation correlation analysis of NR3C1 was performed, and it was found that the methylation type of NR3C1 was mainly m6A methylation; the frequency of methylation hypermutation in the CDS region was also high, and the homologous region and the promoter of the NR3C1 gene showed hypomethylation and hypermethylation differences in the analyses of gastric tissues, races, and gastric cancer subtypes, respectively. Among the methylated peninsular mutations, the TSS200; 5′UTR, 5′UTR, and TSS1500; 5′UTR regions were statistically significant.

**Conclusion:**

NR3C1 can be used as a potential methylation marker of RAM for the prevention and treatment of gastric cancer.

## 1 Introduction

Gastric cancer, a disease with high molecular and phenotypic heterogeneity, is the fifth most common form of cancer and the third leading cause of death worldwide (Smyth et al., 2020). Although the incidence and mortality rates of gastric cancer have decreased in the recent years in various countries, it is still one of the major diseases threatening human health (Yang W. J. et al., 2023). According to epidemiological analyses, stomach cancer is most prevalent in East Asia, followed by Eastern and Central Europe, and it is most prevalent in men (Ló et al., 2023). Currently, *Helicobacter pylori* remains the primary risk factor for gastric cancer, and evidence suggests that alcohol, processed foods, and diets high in salt and fat are also risk factors for gastric cancer (Maddineni et al., 2022). For the time being, rapid advances in the treatment of gastric cancer are dependent on advances in the diagnosis and staging of gastric cancer, genomic classification, surgical resection and treatment, systemic chemotherapy and radiotherapy, and targeted therapies and immunotherapies (Johnston and Beckman, 2019).

Currently, medicinal plants are gaining attention because of their combined food and medicinal value. Several studies have demonstrated the antitumor activity of medicinal plants, and they have been shown to enhance the efficacy of chemotherapy, radiotherapy, targeted therapy, and immunotherapy (Wang S. et al., 2020). Ginseng and ginsenosides possess anti-colon cancer (Zhao et al., 2022) and anti-gastrointestinal tumor (Ni et al., 2022) activities. Similarly, honeysuckle is also used as a medicinal herb, and its extracts showed cytotoxicity in triple-negative breast cancer (Li et al., 2023) and the induction of apoptosis in hepatocellular carcinoma (Zhang et al., 2022) and HeLa cells (Li et al., 2017). Polyphenol extracts from yam are capable of exerting anti-colorectal cancer effects through the NF-κB/p6 and STAT3 signaling pathways (Yang X. et al., 2023). Diosgenin, a constituent of yam, is also a component that exhibits anticancer activity (Ren et al., 2023). Rhizoma Atractylodis Macrocephalae (RAM) is a medicinal plant from the Asteraceae family. Studies have shown that RAM exhibits anti-inflammatory (Jeong et al., 2019), intestinal microflora regulatory (Cheng et al., 2023), and anti-cancer (Wang et al., 2024) activities. Atractylenolide I isolated from RAM ameliorates cancer cachexia via IL-6 and extracellular vesicles and inhibits the STAT3 signaling pathway (Fan et al., 2022). RAM polysaccharide induces antiproliferative activity against glioma C6 cells via the mitochondrial pathway by activating caspase 6/9 and PARP production (Li et al., 2014). The ethanol extract of RAM inhibits gastritis and gastric cancer via the AKT/NF-κB pathway (Amin et al., 2022). Alcohol-soluble polysaccharides from RAM induce apoptosis in esophageal cancer cells (Eca-109) via the mitochondrial pathway (Feng et al., 2019).

Machine learning, which is the process of automatically extracting laws and patterns from data by allowing computers to perform specific tasks, can be used to extract nonlinear and seemingly unrelated factors that are difficult to be detected by traditional methods, leading to more accurate feature selection. Therefore, the use of LASSO regression and support vector machine–recursive feature elimination (SVM-RFE) and random forest to build a model to detect the target can improve the accuracy and confidence of the target and overcome the limitations of previous studies. Network pharmacology transforms TCM research from a “one-target–one-drug” model to a “network-target-multi-component” model from the systemic and molecular level, revealing the association between drug–gene–disease synergistic modules (Li and Zhang, 2013). WGCNA is used to characterize the pattern of association between genes in microarray samples Swimsuit to find genes that are highly associated with disease and to screen for candidate biomarkers as well as therapeutic targets (Langfelder and Horvath, 2008). Relevant studies have shown that WGCNA has been widely used to predict relevant markers, such as the identification of diagnostic markers of immunity and oxidative stress in diabetic nephropathy (Xu et al., 2023) and the identification of biomarkers for breast cancer (Tian et al., 2020).

**FIGURE 1:**
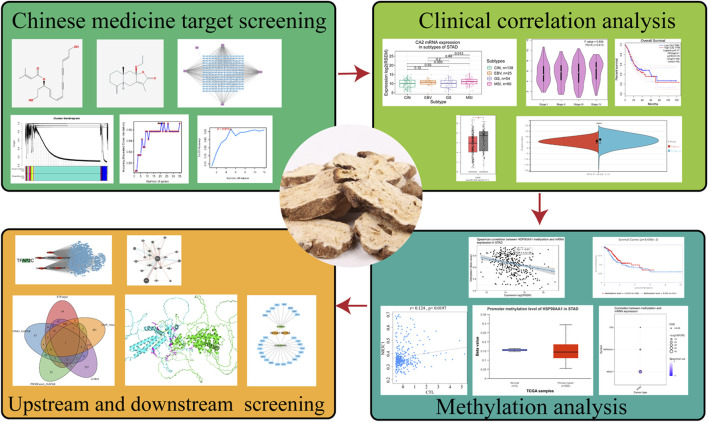
Flowchart.

In this study, we used network pharmacology to initially find the relevant targets of RAM for the treatment of gastric cancer and further screened the most closely related targets of RAM with gastric cancer by WGCNA and a three-group machine learning algorithm. The clinical correlation analysis of these targets was used to identify the core targets, and the related mechanism of RAM in treating gastric cancer was studied in depth. The database was used to screen out the upstream transcription factors of the core target and the possible downstream targets, and the protein with the lowest binding energy was predicted by molecular docking between the two proteins; that is, it is most likely to be the downstream of the core target. It provides new insights and ideas for the treatment of gastric cancer with RAM. The experimental flow of the article is shown in Figure 1.

## 2 Materials and methods

### 2.1 Screening of Chinese herbal medicine active ingredients

The name of the Chinese medicine, Rhizoma Atractylodes Macrocephala, was searched through the Traditional Chinese Medicine Systematic Pharmacology Profiling Platform (TCMSP) database (https://old.tcmsp-e.com/tcmsp.php). In addition, the preliminary active ingredients were screened by the DL value ≥0.18 and OB value ≥30%. The preliminary screening results were used as a basis for further screening of active ingredients on a pharmacokinetic and drug-likeness basis using the SwissADME database (http://www.swissadme.ch/).

### 2.2 Prediction of target genes of Chinese herbal medicine active ingredients

The active ingredients identified in section 2.1 were used to predict target genes using the SwissTargetPrediction (http://www.swissadme.ch/) database, and they were de-emphasized to obtain the final predicted target genes.

### 2.3 Disease database target screening

A search was conducted for gastric cancer-related genes in the GeneCards database (https://previous.genesgards.org/) using “Gastric cancer” as keywords; “Gastric cancer” was used as keywords to search for gastric cancer-related genes in the DisGeNET database (https://disgenet.com/); “Gastric cancer” was used as keywords to search for gastric cancer-related genes in the GEO database (https://www.ncbi.nlm.nih.gov/geo/), and target microarrays were screened for gastric cancer-related genes by |LogFC|>2, p-value < 0.05.

### 2.4 WGCNA analysis

The GEO database screening results were used as the destination matrix, and the weighted gene co-expression network analysis was performed using the Ouyi Cloud platform (https://cloud.oebiotech.com/#/) to screen the modules related to gastric cancer, and the gastric cancer-related genes were obtained.

### 2.5 Acquisition of targets intersecting TCM active ingredients and gastric cancer

The target genes screened in section 2.2 were taken as intersections with the genes associated with gastric cancer in sections 2.3 and 2.4, and the results were visualized using the microbiotics platform (http://www.bioinformatics.com.cn/).

### 2.6 Differential gene analysis

The intersecting genes obtained from section 2.5 were passed through the GEO database to obtain the expression matrix. Clustering heatmap visualization and visualization of the top 10 upregulated genes and all downregulated genes were carried out using the microbiome platform.

### 2.7 Machine learning to further screen hub genes

The hub genes obtained from section 2.5 were plotted using the expression matrix and the results obtained from the three algorithms, namely, LASSO, random forest, and SVM, and they were taken as intersections and visualized using the microbiotics platform. The results obtained from the three algorithms were plotted on a PPI network using Cytoscape 3.9.1.

### 2.8 Clinical correlation analysis of hub genes

Differential expression, graded staging, copy number, and survival curve analyses were performed via the GEPIA website (http://gepia.cancer-pku.cn/). For analysis using the GEPIA database, |Log_2_FC| cutoff = 1 and p-value cutoff = 0.01 were used as positive judgment values. Violin plot analysis of differential expression of target genes was performed via SangerBox (http://www.bioinformatics.com.cn/). In this process, samples with an expression level of zero were filtered, and samples with less than three gastric cancer samples were also excluded. GSEA was performed using the CAMOIP (http://www.camoip.net/) website. Differential expression analysis in different subtypes of gastric cancer was performed using the GSCA (Liu et al., 2023) (https://guolab.wchscu.cn/GSCA/#/) website. In this process, samples with a tumor sample size greater than 10 were selected for analysis. Immunohistochemistry and immunofluorescence analyses of each target gene were performed using the Protein Atlas database (http://www.protein.org/).

### 2.9 Analysis of mutation sites in hub genes

The analysis of mutation sites and mutation subtypes of each target gene was performed using the GSCA website to obtain the waterfall plot of SNV mutation frequency and the bubble plot analysis of the association of the negative mutation variant (CNV) of each target gene in gastric cancer among heterozygotes and heterozygotes. The mutational associations of the driver and hub genes in gastric cancer and MSI expression levels were obtained through CAMOIP database analysis.

### 2.10 Epigenetic regulation of hub genes

Methylation expression level profiles of the hub genes in normal and gastric cancer subtype groups were obtained from TCGA data in the UALCAN database (https://ualcan.path.uab.edu/index.html). The correlation of the methylation levels of hub genes in gastric cancer subtypes with CTL markers and the survival curves of hypermethylated subgroups and hypomethylated subgroups were obtained by selecting Query Gene through the TIDE database (http://tide.dfci.harvard.edu/). Spearman’s correlation of methylation levels of each core target with mRNA expression in gastric cancer and bubble plots were obtained using the GSCA database.

### 2.11 Correlation of hub genes with methylation-related genes

The correlation of the core targets with methylation write-, erase-, and read-related genes in gastric cancer was obtained from the TIMER2 database (http://timer.cistrome.org/) and summarized as a table file, and the correlation heatmap was drawn using the application of ChiPlot (https://www.chiplot.online/).

### 2.12 Relationship of hub genes to methylation mutation types and mutant regions

Mutations in the hub genes were obtained using the RMBase database (https://rna.sysu.edu.cn/rmbase3/modgene.php) group with mammal as an option, genome with *Homo sapiens* as an option, and assembly with hg38 as an option, and they were utilized for microbial letter visualization. The mutated regions of m6A Num, m5C Num, 2′-O-Me Num, and RNA-editing Num were also visualized using microbiotics.

### 2.13 Hub genes and DNA methylation

The EWAS Data Hub database (https://ngdc.cncb.ac.cn/ewas/datahub) was used to obtain methylation of core gene ontologies and promoters in different tissues, the methylation levels of the six major races, differences in methylation levels between patients and healthy samples in gastric cancer, survival curve analysis, and scatterplots of methylation in relation to the expression.

### 2.14 Survival analysis of hub genes with CpG methylation patterns

Univariate and multivariate analyses were performed using the MethSurv database (https://biit.cs.ut.ee/methsurv/) and the Cox risk model based on the methylation level of the patient’s CpG site (probe) according to the region of the hub genes based on the gastric cancer database, which was analyzed by Kaplan–Meier (KM) plots survival differences between hypomethylation and hypermethylation. Cluster analysis of individual CpGs in hub genes was performed using a heatmap format to correlate methylation levels with available patient characteristics and gene subregions. Heatmap methylation levels (1 = completely methylated; 0 = completely unmethylated) are shown as continuous variables from blue to red. Rows correspond to CpG, and columns correspond to patients. The factors covered include age, survival status, and ethnicity, among others.

### 2.15 Molecular docking of hub genes with active ingredients in traditional Chinese medicine

Based on the hub genes screened out, their corresponding active ingredients were confirmed according to section 2.2. The PDB database (https://www.rcsb.org/) was used to download the PDB file format of the corresponding proteins and imported into PyMOL software to remove water molecules and ligands. The mol2 file format of the corresponding active ingredient was downloaded from the TCMSP database, imported into AutoDock Tools 4.2.0 for molecular docking, and visualized using PyMOL.

### 2.16 Screening of upstream transcription factors and downstream proteins of hub genes

The hTFtarget database (http://bioinfo.life.hust.edu.cn/hTFtarget), the ChIP-Atlas database (https://chip-atlas.org/), the JASPAR database (https://jaspar2022.genereg.net/docs/), the PWNEnrich database (https://www.bioconductor.org/packages/release/bioc/html/PWMEnrich.html), and the GTRD database (http://gtrd20-06.biouml.org/) were used to obtain the upstream transcription factors of the hub genes and visualize them by taking the intersections by microbial letters and by using Cytoscape 3.9.1. Downstream target proteins of the hub genes were obtained based on the STRING database ((https://cn.string-db.org/) and GeneMANIA database (http://genemania.org/) and visualized by microbiological signaling intersections and by using Cytoscape 3.9.1.

### 2.17 Molecular docking between hub genes and downstream target proteins

According to the UniProt database (https://www.uniprot.org/), we obtained the entry numbers of the hub genes and downstream proteins and imported them into the AlphaFold Protein Structure Database (https://alphafold.com/) to download the corresponding PDB files. We imported the corresponding PDB file into the GRAMM database (https://gramm.compbio.ku.edu/) for molecular docking and viewed the alignment table for visualization using PyMOL software application.

## 3 Results

### 3.1 Screening of Chinese herbal medicine active ingredients

According to the TCMSP database, a total of 55 active ingredients of RAM were obtained, and seven active ingredients were screened based on the DL value ≥0.18 and OB value ≥30%. Then, the heavy species of active ingredients were screened on the basis of pharmacokinetic and drug-likeness. A total of five ingredients were found to satisfy the high absorption of GI absorption and the presence of more than two ”yes” in drug-likeness, so all five active ingredients were confirmed as preferred active ingredients (Table 1).

**TABLE 1:** Active components of Atractylodes macrocephala.

Mol ID	Molecule name	OB (%)	DL
MOL00072	8β-Ethoxy atractylenolide Ⅲ	35.95	0.21
MOL00049	3β-Acetoxyatractylone	54.07	0.22
MOL00020	12-Senecioyl-2E,8E,10E-atractylentriol	62.40	0.22
MOL00022	14-Acetyl-12-senecioyl-2E,8E,10E-atractylentriol	63.37	0.30
MOL00021	14-Acetyl-12-senecioyl-2E,8E,10E-atractylentriol	60.31	0.31

### 3.2 Prediction of target genes of Chinese herbal medicine active ingredients

**FIGURE 2:**
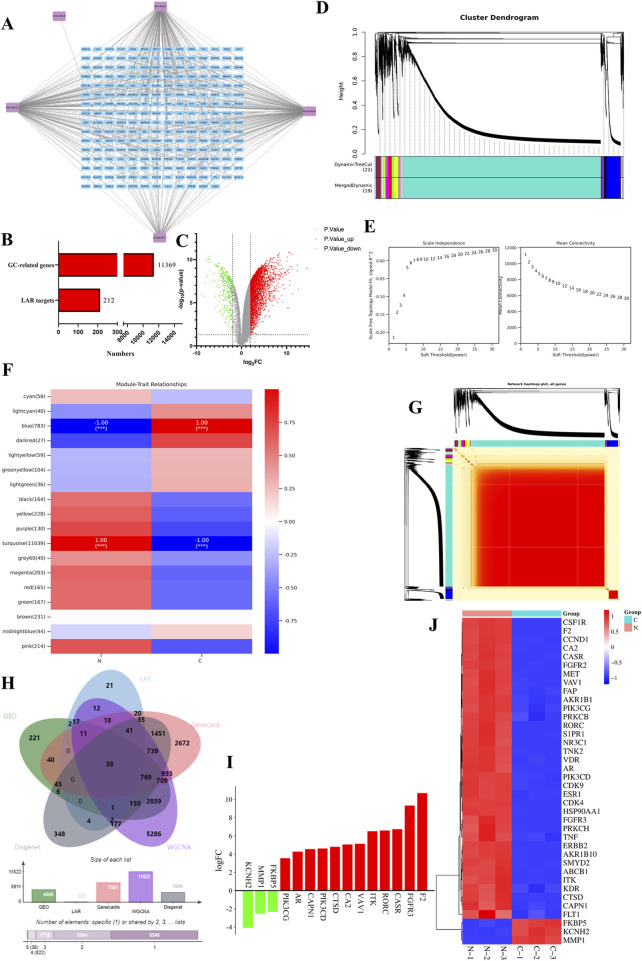
Targets of bitter almond active ingredients and differentially expressed genes in gastric cancer. **(A)** Network diagram of bitter almond active ingredient-targets. Purple color represents the ingredients of bitter almonds, and blue color represents all the targets of active ingredients. **(B)** All genes of gastric cancer and the number of targets of bitter almond active ingredients. **(C)** Volcano plot of differentially expressed genes of GSE49051, where orange represents downregulated genes, green represents upregulated genes, and gray indicates no difference or no significance. **(D)** Cluster dendrogram of WGCNA analysis. **(E)** Left figure is the correlation coefficient corresponding to different power, and the right figure is the average connection degree of the network constructed by different power values. **(F)** Vertical axis represents each module, the horizontal axis represents each trait, and the correlation between modules and traits is shown in the results, with positive correlation in red and negative correlation in green. **(G)** Heatmap of all gene clustering. **(H)** Intersection plot of bitter almond and gastric cancer targets. Green and blue represent gastric cancer and bitter almond, and pink and yellow represent the GEO and WGCNA datasets, respectively. **(I)** The top 10 upregulated and downregulated genes in the intersection target list. **(J)** Heatmap of intersecting targets. Pink and green represent the tumor group and normal group, respectively.

Based on the above five active ingredients and the SwissTargetPrediction database, a total of 376 target genes were obtained, which were de-emphasized, resulting in 212 target genes (Figure 2A).

### 3.3 Disease database target screening

A total of 11,369 gastric cancer-related genes were obtained after taking the concatenated set by the GeneCards database, DisGeNET database, and GES49051 gene chip from the GEO database, and the histogram is as follows (Figures 2B,C).

### 3.4 WGCNA analysis

Weighted gene co-expression network analysis of the expression matrix of gastric cancer-related genes by the Ouyi Cloud platform showed that among the 18 color modules, the blue and turquoise modules had the highest correlation with gastric cancer, with a total of 11,822 genes (Figures 2D–G).

### 3.5 Acquisition of targets intersecting TCM active ingredients and gastric cancer

The gastric cancer-related genes obtained from the database were intersected with the genes in the module with the highest correlation with gastric cancer in WGCNA and the target genes of the active ingredients of traditional Chinese medicines, and a total of 38 intersected genes were obtained (Figure 2H).

### 3.6 Differential gene analysis

The expression matrix of the 38 intersecting genes was analyzed by clustering heatmap, and a total of 35 upregulated genes were found, such as *CSF1R*, *F2*, and *CCND1*. The downregulated genes were *MMP1*, *KCNH2*, and *FKBP5* (Figures 2I, J).

### 3.7 Machine learning algorithms to further screen hub genes

**FIGURE 3:**
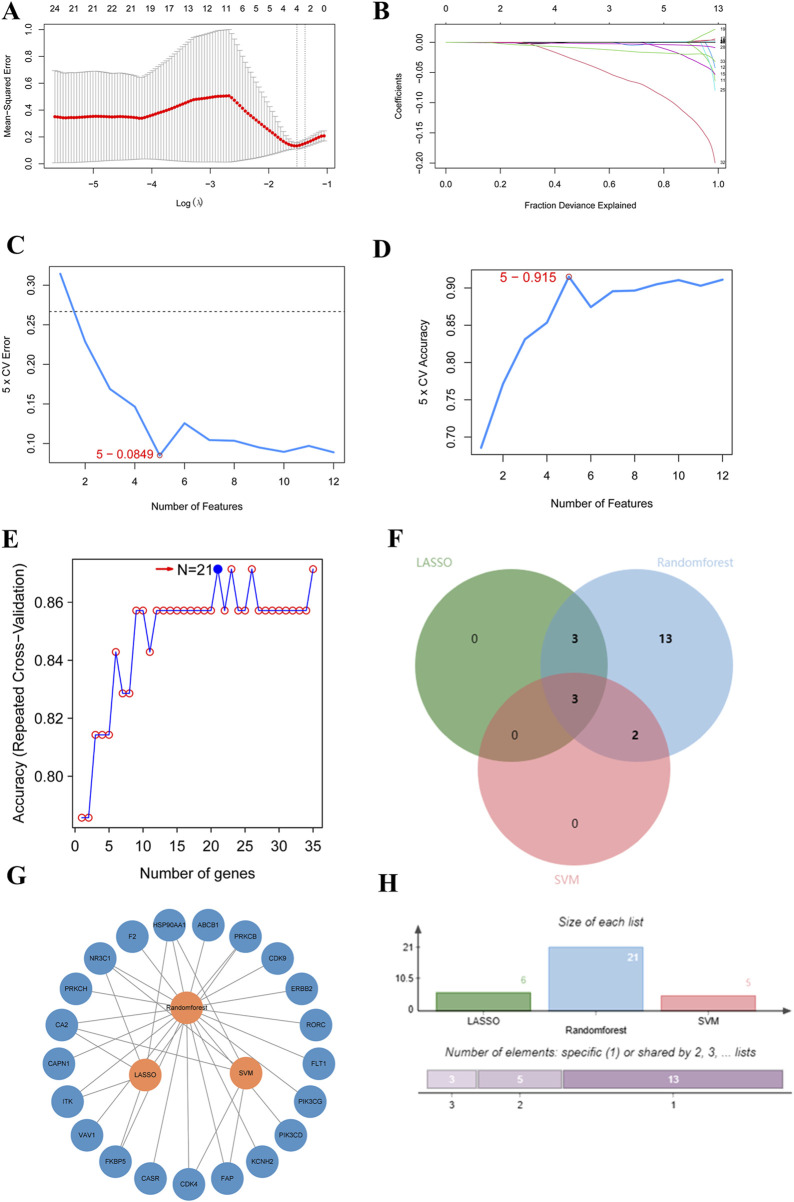
Core target prediction in R **(A–E)**. Key gene prediction. **(A,B)** Data obtained from the LASSO algorithm. **(C,D)** Data obtained from Support Vector Machine (SVM). **(E)** Data obtained from Random Forest. **(F)** LASSO target intersection plot. Green and blue represent Random Forest and Support Vector Machine respectively, with pink denoting LASSO. **(G)** Target intersection network diagram. **(H)** LASSO target intersection histogram.

According to the three algorithms, namely, LASSO, random forest, and SVM in the machine algorithm, the hub genes obtained were 6, 21, and 5, respectively (Figures 3A–E). After taking the intersection, CA2, NR3C1, and HSP90AA1 were found to be their common targets (Figures 3F–H).

### 3.8 Clinical relevance analysis

**FIGURE 4:**
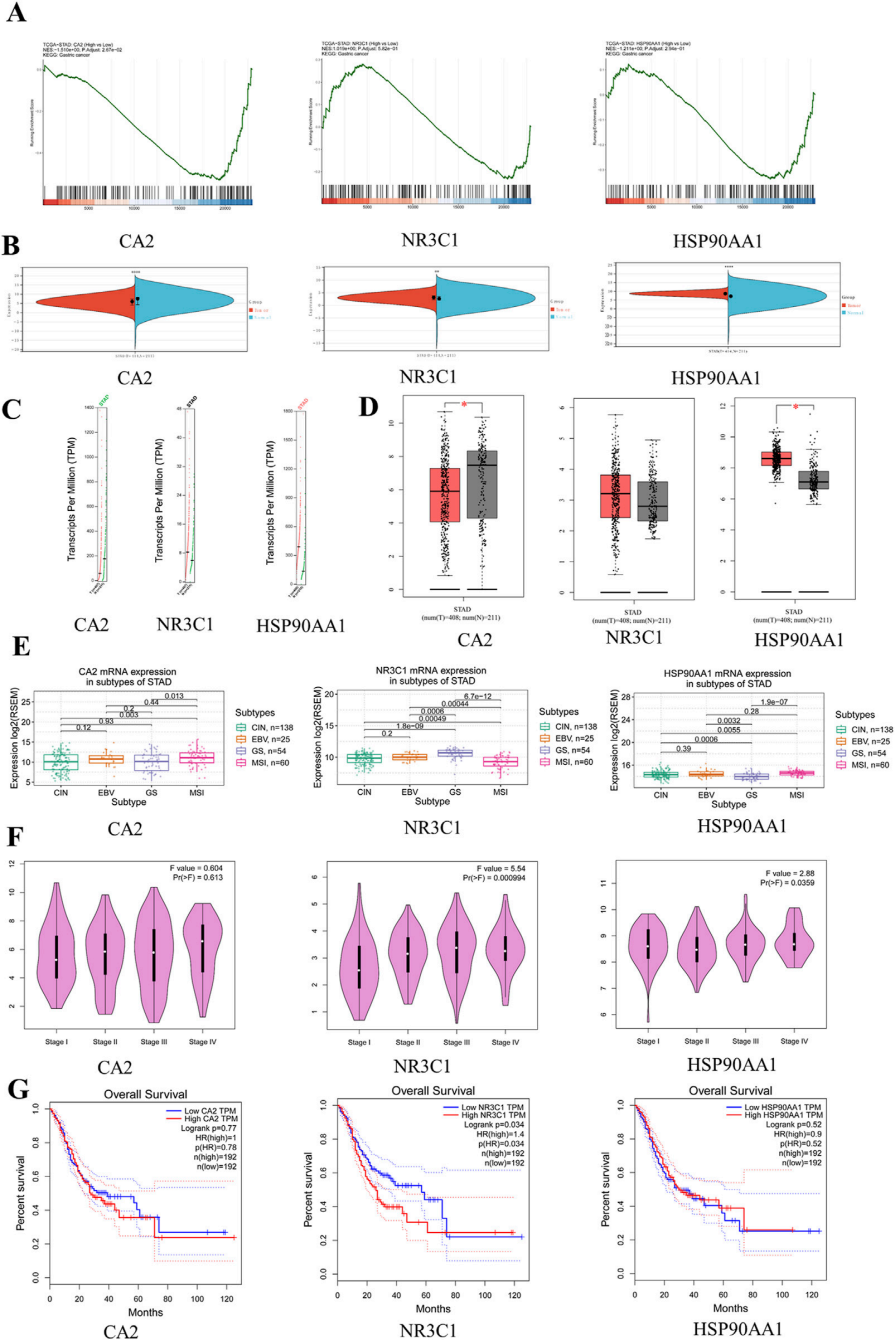
Clinical correlation analysis of hub genes. **(A)** GSEA graph of hub genes. **(B)** Expression levels of hub gene mRNA. Red represents the tumor group, and green represents the normal group. **(C)** Expression levels of hub genes copy number. Red represents the tumor group, and green represents the normal group. **(D)** Expression level of hub gene protein. **(E)** Expression levels of hub genes in gastric cancer subtypes. **(F)** Correlation between hub genes and clinical stage of gastric cancer. **(G)** Survival curve diagram of hub genes. The horizontal coordinate represents the survival time.

**FIGURE 5:**
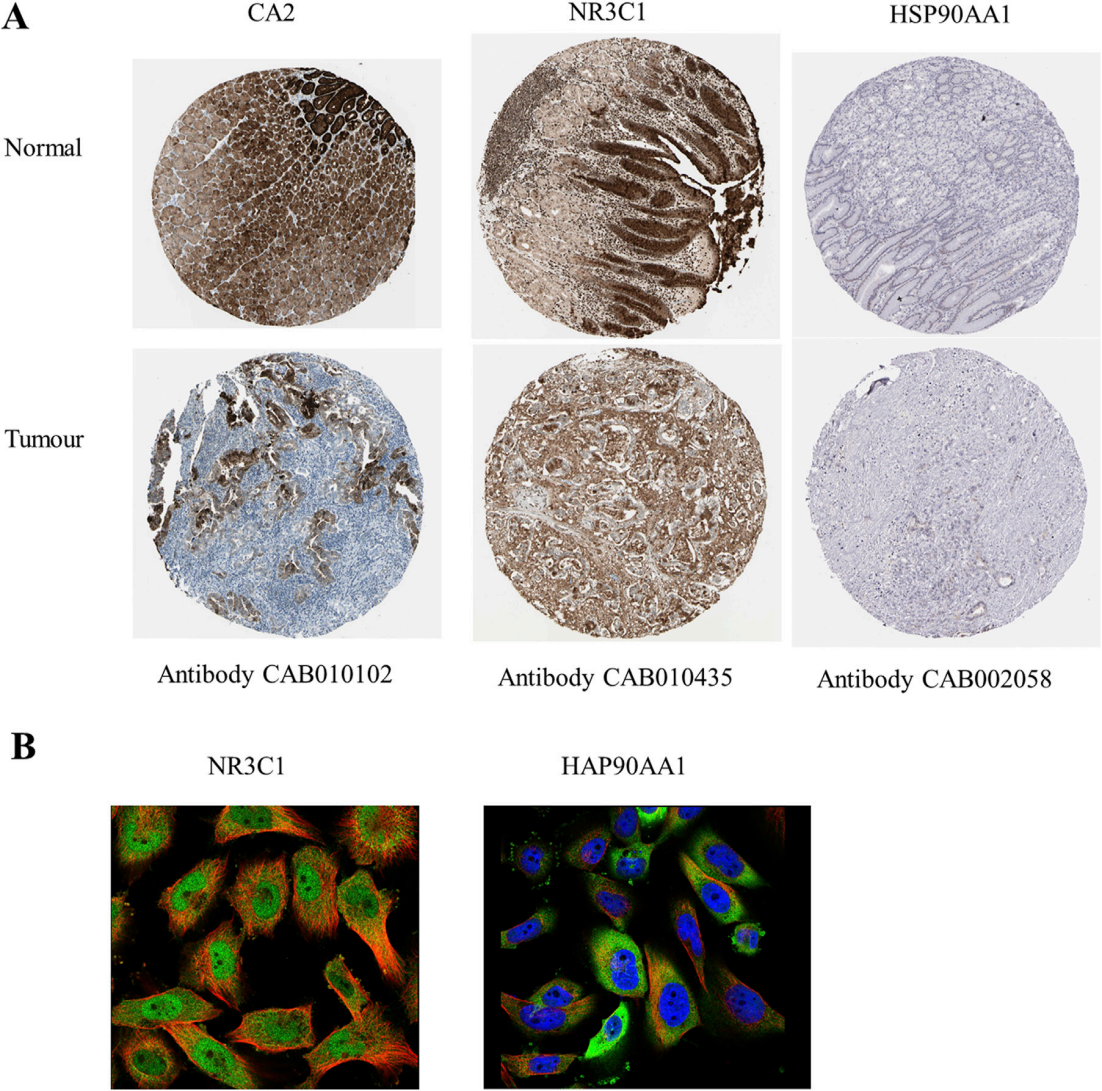
Expression and prognostic analysis of hub genes. **(A)** Immunohistochemistry of hub genes in normal gastric tissue and gastric adenocarcinoma tissue. Brown color shows the expression level of hub genes. **(B)** Fluorescence localization map of hub genes in tumor tissues. Blue represents the nucleus, red represents microtubule organization, and green represents hub genes.

Clinical correlation analysis of CA2, NR3C1, and HSP90AA1 revealed that only CA2 was statistically significant (p = 0.0267) in the analysis of the KEGG pathway in gastric cancer (Figure 4A), and in the analysis of TCGA gastric cancer samples (tumor = 414, normal = 211), CA2 (p < 0.0001), NR3C1 (p < 0.01), and HSP90AA1 (p < 0.0001) were significant (Figure 4B). Analysis by the GEPIA database revealed (tumor = 408, normal = 211) that CA2 and HSP90AA1 were differentially expressed in gastric cancer, CA2 (p < 0.05) was lowly expressed in gastric cancer, and HSP90AA1 (p < 0.05) was highly expressed in gastric cancer (Figures 4C, D). In the correlation analysis of grading and staging, NR3C1 (p = 0.000994) and HSP90AA1 (p = 0.0359) showed significant differences (Figure 4F). In addition, the level of NR3C1 expression (p = 0.034) also significantly affected the survival time of the patients (Figure 4G). Among different subtypes of gastric cancer, CA2 mRNA was significantly different in CIN and MSI (p = 0.003) and GS and MSI (p = 0.013). nR3C1 mRNA was significantly different in CIN and GS (p = 1.8e-09), CIN and MSI (p = 0.00049), EBV and GS (p = 0.0006), EBV and MSI (p = 0.00044), and GS and MSI (p = 6.7e-12). HSP90AA1 mRNA was significantly different in CIN and GS (p = 0.0006), CIN and MSI (p = 0.0055), EBV and GS (p = 0.0032), and GS and MSI (p = 1.9e-07) (Figure 4E). According to the immunohistochemistry results, it was found that CA2 showed a low expression status in the tumor group, which was consistent with the results of the GEPIA database analysis (Figure 5A). According to the immunofluorescence results, it was found that NR3C1 was mainly expressed in the nucleus and HSP90AA1 was mainly expressed in the cytoplasm (Figure 5B).

### 3.9 Analysis of mutation sites in hub genes

**FIGURE 6:**
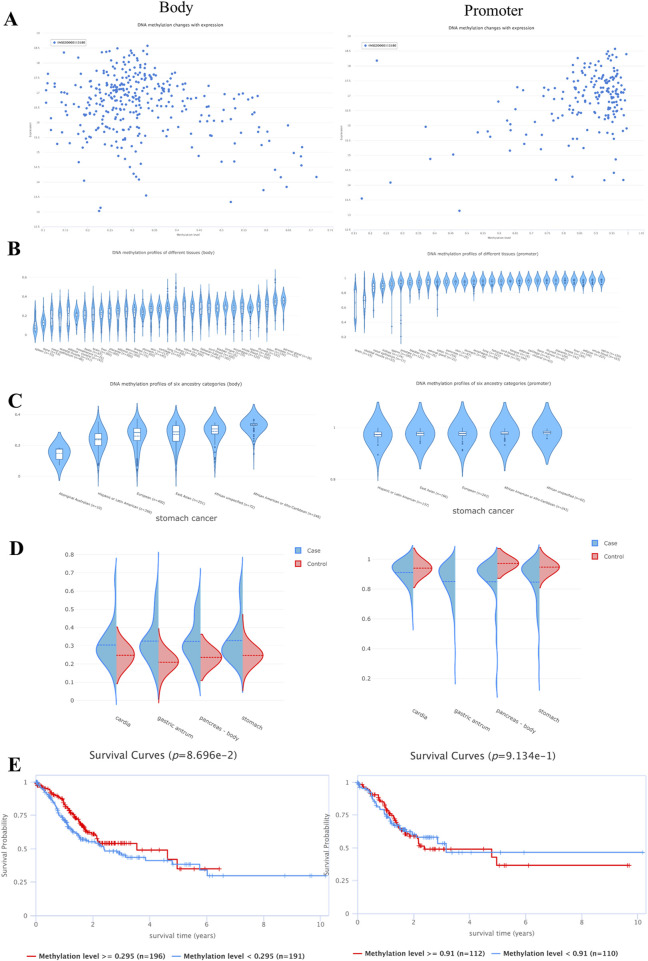
Effect of mutations in the hub genes on gastric cancer. **(A)** SNV mutation sites and types of MET. The circle color represents the mutation type, and the line length represents the mutation frequency. **(B)** Waterfall plot of SNV mutation frequency of hub genes. The upper bar indicates the proportion of hub gene mutations in the 51 samples. **(C)** Bubble plots of heterozygous and pure heterozygous CNV mutations in the hub genes. The larger the bubble, the higher the proportion of mutations. **(D)** Mutational associations of driver genes with the hub genes. **(E)** Box plot of MSI expression levels of hub genes.

According to the GSCA database, the somatic mutation rate of CA2 was found to be 1.14%, the somatic mutation rate of NR3C1 was found to be 2.28%, and the somatic mutation rate of HSP90AA1 was found to be 2.28%, in which CA2 mainly had missense mutation and base deletion, NR3C1 mainly had missense mutation and a nonsense mutation, and HSP90AA1 mainly had missense mutation with in-frame deletion mutation. Among them, NR3C1 had nonsense mutation in gastric cancer (Figures 6A, B). CA2 had the largest percentage of heterozygous amplification, and NR3C1 and HSP90AA1 had the largest percentage of heterozygous deletion; CA2 had the largest percentage of pure heterozygous amplification, and only HSP90AA1 had a certain percentage of pure heterozygous deletion (Figure 6C). In the CA2 high-expression group compared with the low-expression group, there were no statistical differences in some typical driver genes. Whereas NR3C1 had differences in driver genes such as TTN (p < 0.0001), TP53 (p < 0.05), MUC16 (p < 0.05), and SYNE1 (p < 0.001), HSP90AA1 had differences in TTN (p < 0.05), CSMD3 (p < 0.01), FAT4 (p < 0.05), and KMT2D (p < 0.01), which had more significant differences (Figure 6D). The MSI expression levels of NR3C1 and HSP90AA1 were significantly different in the high-expression group versus the low-expression group, with NR3C1 having higher MSI expression in the low-level group (p < 0.0001), whereas HSP90AA1 had higher MSI expression in the high-level group (p < 0.0001) (Figure 6E).

### 3.10 Epigenetic regulation of hub genes

**FIGURE 7:**
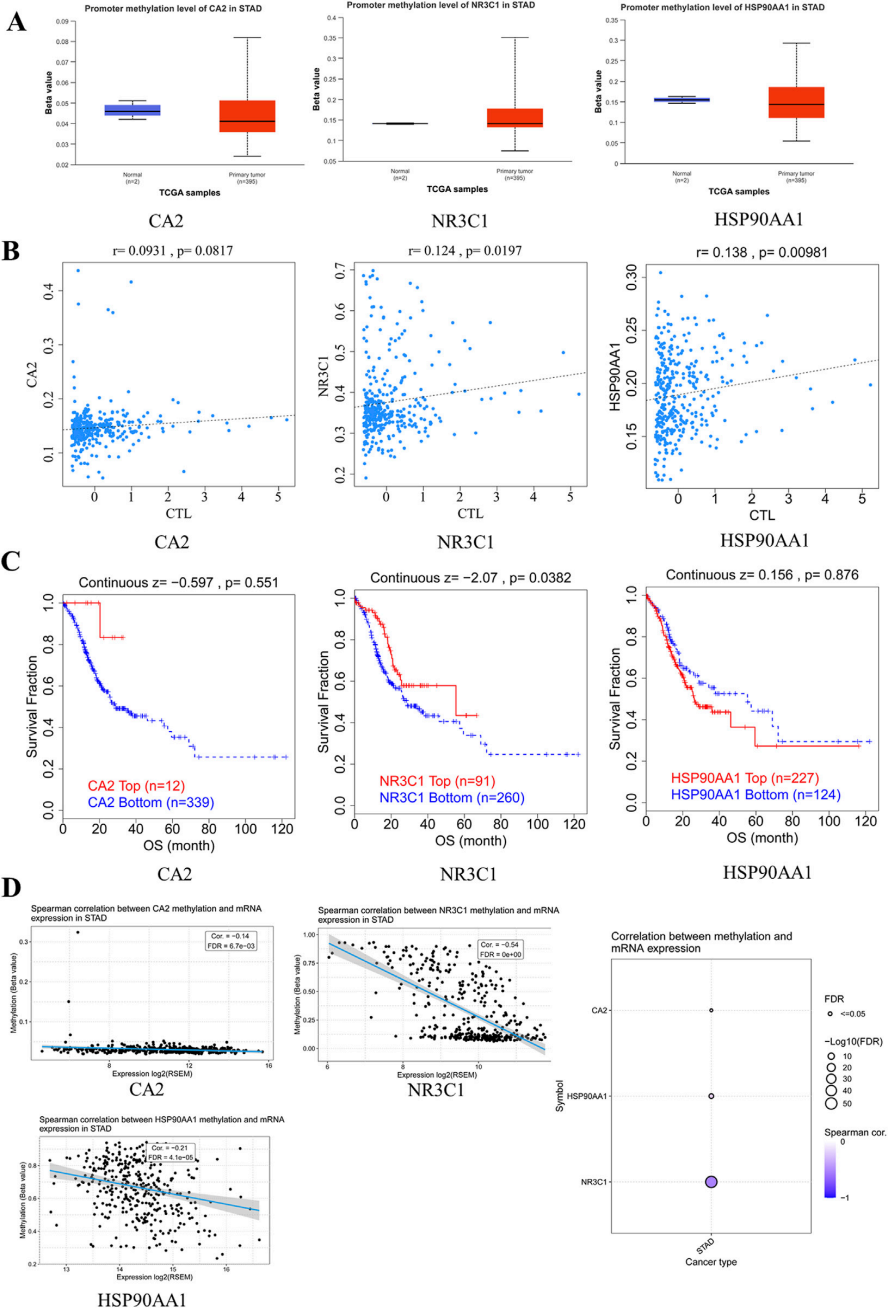
Hub genes are involved in epigenetic regulation. **(A)** Expression level map of hub gene methylation. Blue and red represent the normal and tumor groups, respectively. **(B)** Correlation between hub gene methylation levels and CTL markers. **(C)** Survival curves of hypermethylated and hypomethylated subgroups of hub genes were plotted. **(D)** Expression of core target methylated mRNA in STAD.

None of the promoter methylation expression levels of CA2, NR3C1, and HSP90AA1 were significantly different in gastric cancer (Figure 7A). NR3C1 (p = 0.0197) and HSP90AA1 (p = 0.00981) had a strong correlation with CTL markers and were significantly different (Figure 7B). Survival analysis of patients with high versus low methylation levels found only NR3C1 to be statistically significant (p = 0.0382) (Figure 7C). Spearman’s correlation showed that NR3C1 methylation had one of the strongest negative correlations with its mRNA expression and FDR = 0.00 (Figure 7D).

### 3.11 Correlation of hub genes with methylation-related genes

**FIGURE 8:**
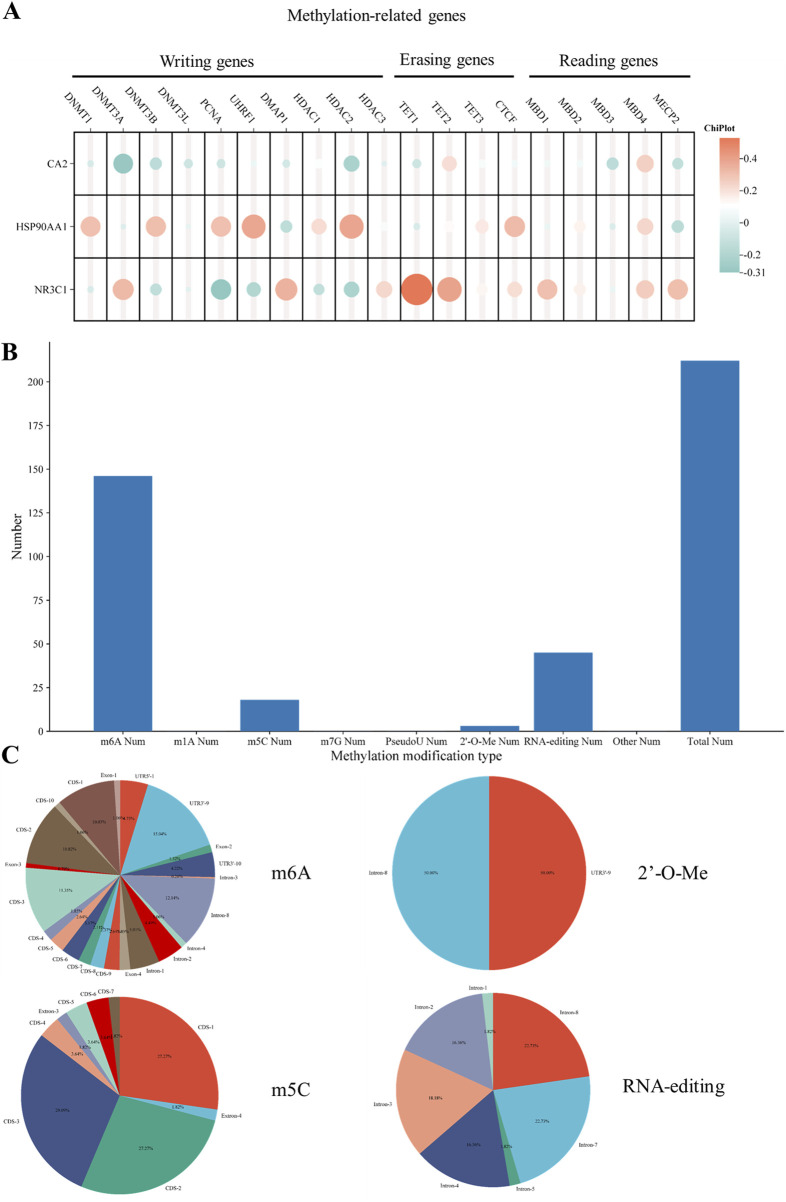
Hub gene methylation correlation analysis **(A)**. Correlation between hub genes and methylation-related genes **(B)**. NR3C1 methylation type number histogram **(C)**. Frequency pie chart of mutation sites in each methylation type.

Among the methylation transferase DNMT family, DNM1 and DNMT3B were strongly correlated with HSP90AA1 and DNMT3A was strongly correlated with NR3C1. Among the genes related to methylation, PCNA, UHRF1, and HDAC2 were all strongly correlated with HSP90AA1, whereas DMAP1 was strongly correlated with NR3C1. Among the erasure-related genes, TE11 and TET2 were strongly correlated with NR3C1 (Figure 8A).

### 3.12 Relationship of hub genes with methylation mutation types and mutant regions

Based on the histogram statistics of relevant mutation types, NR3C1 methylation was mainly in m6A, m5C, 2′-O-Me, and RNA-editing mutations (Figure 8B). According to the frequency map of the mutated regions, among the m6A mutations, the highest mutation frequency was mainly in the four regions of CDS-1, CDS-3, intron-8, and UTR3′-9, which were 10.03%, 11.35%, 12.14%, and 15.04%, respectively. As for the m5C mutation types, the mutations were mainly concentrated in the CDS region, including CDS-1, CDS-2, and CDS-3, with mutation frequencies of 27.27%, 27.27%, and 29.09%, respectively. Among the 2‘-O-Me mutations, the main mutations were in the intron-8 and UTR3’-9 regions, both with a frequency of 50%. Among the RNA-editing mutations, the mutated regions were all in the intron regions, mainly intron-2, 3, 4, 7, and 8. The mutation frequencies were 16.36%, 18.18%, 16.36%, 22.73%, and 22.73%, respectively (Figure 8C).

### 3.13 Hub genes and DNA methylation

**FIGURE 9:**
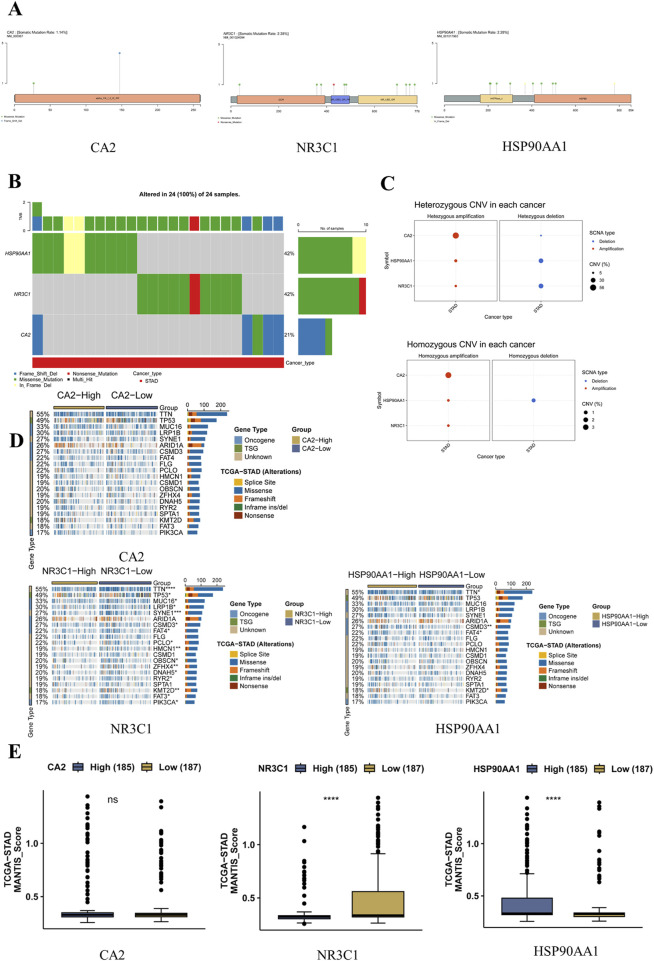
NR3C1 methylation correlation analysis **(A)**. Relationship between gene ontology and promoter methylation levels and DNA expression **(B)**. Methylation expression levels of gene ontology and promoter in various tissues **(C)**. Methylation expression of gene ontology and promoter is different in different species **(D)**. Methylation expression of gene ontology and promoter is different in different types of gastric cancer **(E)**. Survival curves of high and low methylation of gene body and promoter.

The scatterplot of the methylation level and mRNA expression of NR3C1 gene ontology and promoter showed that NR3C1 gene ontology was more densely and highly expressed at a low methylation level; on the contrary, the NR3C1 promoter was at a high methylation level type with a high mRNA expression level (Figure 9A). Through the methylation of NR3C1 gene ontology and promoter in different tissues, it can be seen that the NR3C1 gene ontology is at a low level of methylation in gastric tissues, whereas the NR3C1 promoter is at a high level of methylation in gastric tissues (Figure 9B). The methylation level of NR3C1 is at a low level of methylation among the six major ethnic groups, among which Aboriginal Australian methylation levels were the lowest and the highest levels were in African American or Afro-Caribbean. The NR3C1 promoter, on the other hand, was hypermethylated in all five major ethnic groups (Figure 9C). The NR3C1 gene ontology was hypomethylated in all four portions of the stomach, and the tumor group had higher methylation levels than the control group in all four portions of the stomach. The NR3C1 promoter, on the other hand, was at high methylation levels in all four parts of the gastric tissue, and except for the gastric antrum (gastric sinus), the methylation levels were higher in the control group than in the gastric cancer group in the remaining three parts (Figure 9D). The NR3C1 gene ontology was at high and low methylation with a boundary of 0.295, and for the high and low methylation survival analysis, the survival time of hypermethylation was significantly shorter than that of hypomethylation, but the hypomethylation level and hypermethylation level survival curves were not statistically significant (p = 0.08696), whereas the NR3C1 promoter was divided into high and low methylation with the boundary of 0.91, and the results showed that the survival time of both high and low methylation of the NR3C1 promoter were approximately 10 years, and there was no statistical significance (p = 0.9134) (Figure 9E).

### 3.14 Survival analysis of hub genes with CpG methylation patterns

Survival time in the survival curves for both hypermethylation and hypomethylation was shown to be 3,500 days for all six mutant regions of NR3C1, with a total of 16 CpG probes, and in the TSS200; 5′UTR region, the three probes cg00629244 (p = 0.00048), cg11152298 (p = 0.0095), and cg18019515 (p = 0.011) were all statistically significant, and all three had a higher likelihood of survival at high methylation levels than at low methylation levels (S2A). In the 5′UTR region, cg06521673 (p = 0.033) and cg17617527 (p = 7.9e-05) were statistically significant in both probes (S2B). In the TSS1500; 5′UTR region, all three probes, that is, cg10847032 (p = 0.00098), cg16335926 (p = 0.017), and cg21702128 (p = 0.0051), were statistically significant (S2C). In the 5‘UTR; TSS1500 region, the first exon; 5’UTR region, the TSS1500; 5′UTR; TSS200 regions, and the above three gene regions, a total of eight gene probes were not statistically significant (S2D-G).

### 3.15 Molecular docking of hub genes with active ingredients in traditional Chinese medicine

The molecular docking results showed that the docking sites of NR3C1 and MOL00021 were HIS-453, YTR-478, and ASN-461, with a docking energy of −2.74 kcal/mol (S3A). The docking site of NR3C1 and MOL00022 was TYR-455, with a docking energy of −0.22 kcal/mol (S3B). Based on the docking energy comparison, NR3C1 was found to dock best with MOL00021 (S3C).

### 3.16 Screening of upstream transcription factors and downstream proteins of hub genes

Transcription factor prediction for NR3C1 was performed by five databases, namely, hTFtarget, FIMO_JASPAR, PWMEnrich_JASPAR, GTRD, and ChIP_Atlas, and the prediction cases were 194, 155, 94, 590, and 515 (S4A), respectively. After taking the intersection, it was found to have a common intersecting transcription factor, TFAP2C, and the intersection situation was visualized (S4B). The downstream proteins were predicted by the STRING database and GeneMANIA, and it was found that both predictions had the same downstream proteins, NCOA1 and NCOA2 (S4C, D).

### 3.17 Molecular docking between hub genes and downstream target proteins

Based on molecular docking of NR3C1 with the downstream predicted proteins NCOA1 and NCOA2, it was found that NR3C1 has multiple binding sites with NCOA1, and the number of binding sites is redundant with that of NR3C1 with NCOA2 (S4E, F). The docking energy of NR3C1 with NCOA1 was −51.2 kcal/mol, which is much larger than that of NR3C1. The docking energy with NCOA2 was −27.6 kcal/mol (S4G).

## 4 Conclusion

**FIGURE 10:**
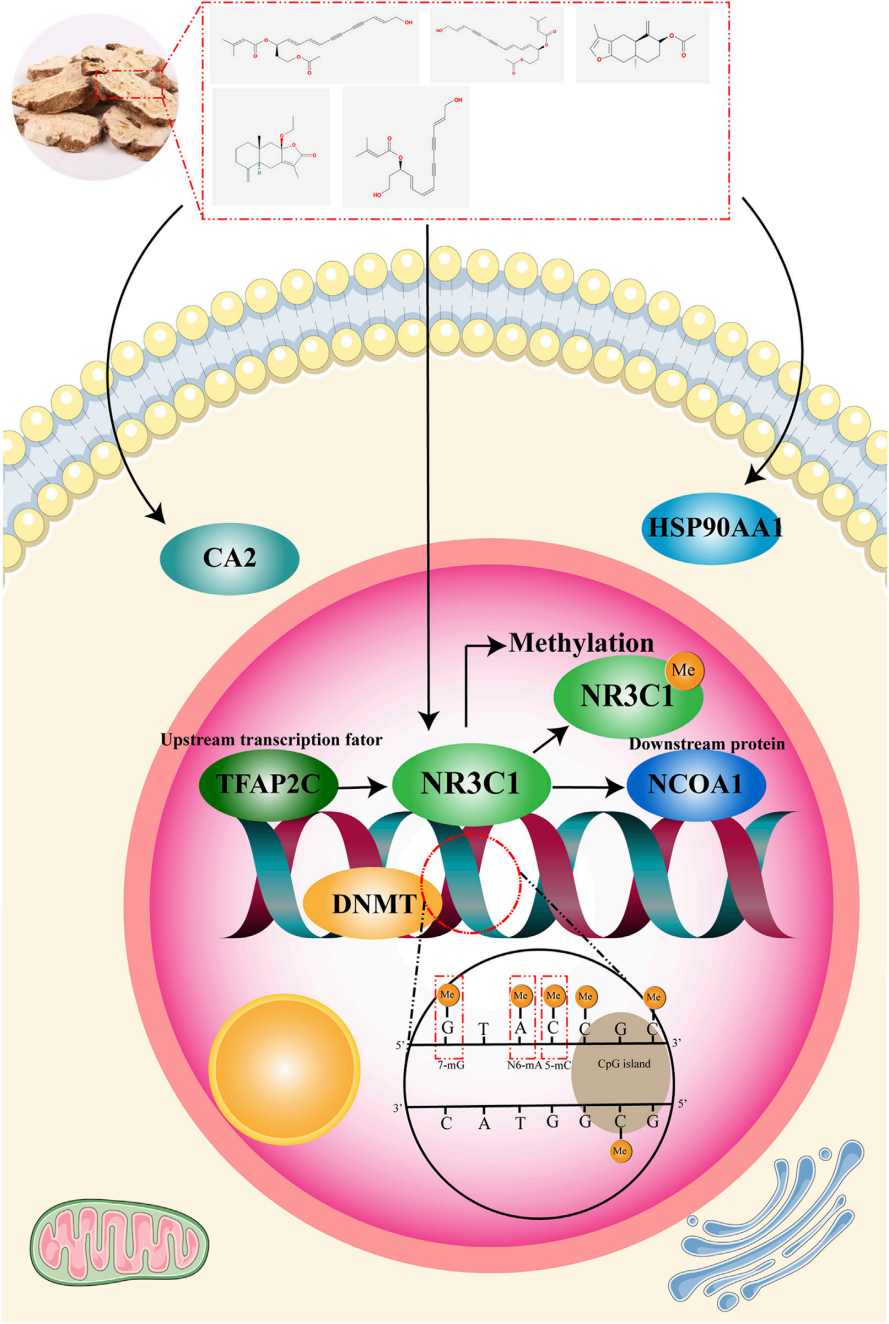
Graphical abstract.

In this experiment, WGCNA and machine learning algorithms were used to predict CA2, HSP90AA1, and NR3C1, the core targets of white Atractylodes against gastric cancer. Clinical correlation and epigenetic regulation analyses revealed that the methylation of NR3C1 was highly correlated with mRNA expression in gastric cancer and that the correlation between NR3C1 and methylated erasure genes, the TET family, was high. This suggests that NR3C1 may be involved in the regulation of methylation. NR3C1 was identified as a potential methylation marker of *Atractylodes macrocephala* against gastric cancer (Figure 10). TFAP2C was screened through multiple databases for possible upstream transcription factors of NR3C1 and possible downstream proteins NCOA1 and NCOA2. Molecular docking revealed that NR3C1 has a lower docking energy with NCOA1, so NCOA1 is more likely to be a downstream protein target of NR3C1.

NR3C1, a gene encoding the glucocorticoid receptor, acts as a regulator of several transcription factors. NR3C1 regulates the downstream transcription factor NRF2 and inhibits human mammary cell proliferation and autophagy, and it promotes dendritic cell differentiation and maturation (Xiong et al., 2021). NR3C1 can act as a direct target of ginsenoside Rg5 to regulate the expression levels of HSPB1 and NCOA4 and inhibit glioblastoma progression (Zhang et al., 2024). Meanwhile, NR3C1, as a transcription factor, is also regulated by a variety of genes. CPEB3 regulates the translation of NR3C1 to restore stress disorder in mice (Lu et al., 2021). Meanwhile, NR3C1 methylation can be involved in a variety of diseases as it is highly correlated with methylation. NR3C1 methylation balances autophagy in pancreatic β-cells and affects impaired insulin secretion and poor glucose tolerance (Wu et al., 2023). Methylation of NR3C1 is associated with alterations in the hypothalamic–pituitary–adrenal axis in schizophrenic patients (Misiak et al., 2021). DNMT1-induced NR3C1 hypermethylation is associated with colorectal cancer cells, and NR3C1 restoration inhibits pro-angiogenic effects on the cells (Zhai et al., 2024).

TFAP2C (activating enhancer-binding protein 2γ) is mainly distributed in the nucleus and functions in the regulation of transcription and cellular signaling. TFAP2C regulates c-Myc to inhibit apoptosis and promotes somatic cell reprogramming through EMT (Wang Y. et al., 2020). TEAD17 is also an important downstream target of TFAP2C, and TEAD17 transcription can activate Th1 and Th4 cells and exacerbate inflammation (Zhang et al., 2023; Zhang et al., 2025). TFAP2C has also been extensively studied in tumors. For example, TFAP2C regulates TGFBR2 transcription and promotes lung tumorigenesis and EMT (Kim et al., 2016), and TFAP2C regulation of CST1 transcriptional activation promotes breast cancer progression and inhibits iron death (Yuan et al., 2024). Nuclear receptor coactivator activator protein 1 (NCOA1) has a variety of biological activities. NCOA1 is a coactivator necessary for the transcriptional activity of STAT6 and is able to mediate the transcription of STAT6 (Lee et al., 2017). At the same time, DNMT1 is also able to regulate methylation and, thus, the overexpression of NCOA1, which triggers myocardial dysfunction (Peng et al., 2022). In summary, we found that TFAP2C–NR3C1–NCOA1 could be a possible pathway for the treatment of tumors.

In this study, the advantage of WGCNA module analysis and the advantage of the high accuracy of the machine learning algorithm predicted that RAM may prevent gastric cancer through CA2, HSP90AA1, and NR3C1, which provides new drugs and targets for the prevention and treatment of gastric cancer, but there is no clinical and experimental verification. RAM, as a medicinal plant with high efficiency and low toxicity, has been rarely studied in the treatment of tumors, and its specific mechanism in gastric cancer is not clear. Follow-up studies are needed to validate the efficacy of RAM for gastric cancer through clinical, cellular, and animal experiments; our screened methylation biomarker NR3C1 should be analyzed further; the reliability of the target should be validated through gene silencing and other means; and the target should be analyzed through immunoprecipitation and other related techniques. Immunoprecipitation and other related techniques can be utilized to analyze the interactions between NR3C1 and NCOA1; dual luciferase and ChIP and EMSA can be utilized to explore the relationship between the transcription factor TFAP2C and NR3C1 to increase the credibility of this study.

## Data Availability

The original contributions presented in the study are included in the article/Supplementary Material, further inquiries can be directed to the corresponding authors.

## Supplementary material

SUPPLEMENTARY FIGURE S1 PCA analysis of the GEO dataset.

SUPPLEMENTARY FIGURE S2 NR3C1 methylation mutation site probe high and low methylation survival curve. **(A–F)** NR3C1 methylation survival curve of probe high and low methylation levels at each mutation site. **(G)** Heatmap of expression difference of NR3C1 methylation mutation sites in probe age, race, etc.

SUPPLEMENTARY FIGURE S3 Molecular docking of drugs with hub genes. **(A)** NR3C1 and MOL000021 molecular docking visualization. **(B)** NR3C1 and MOL000022 molecular docking visualization. **(C)** Molecular docking energy.

SUPPLEMENTARY FIGURE S4 Upstream and downstream screening and molecular docking. **(A)** Screening of upstream targets. **(B)** Visualization of upstream transcription factor screening. **(C)** Screening of downstream targets. **(D)** Visualization of downstream transcription factor screening. **(E)** NR3C1 and NCOA1 molecular docking visualization. **(F)** NR3C1 and NCOA2 molecular docking visualization. **(G)** Molecular docking energy.

SUPPLEMENTARY DATA SHEET S1 Screening of active ingredients and corresponding targets of Rhizoma Atractylodis Macrocephalae.

SUPPLEMENTARY DATA SHEET S2 Analysis results of LASSO, random forest, and SVM.
