# Supplementary figures and images for: Prediction of NR3C1 as a methylation marker for the prevention and treatment of gastric cancer in Rhizoma Atractylodis Macrocephalae based on machine learning algorithm and bioinformatics analysis
Source: Front Genet. 2025 Sep 9;16:1584986. doi: 10.3389/fgene.2025.1584986 (PMC12455331; doi:10.3389/fgene.2025.1584986)

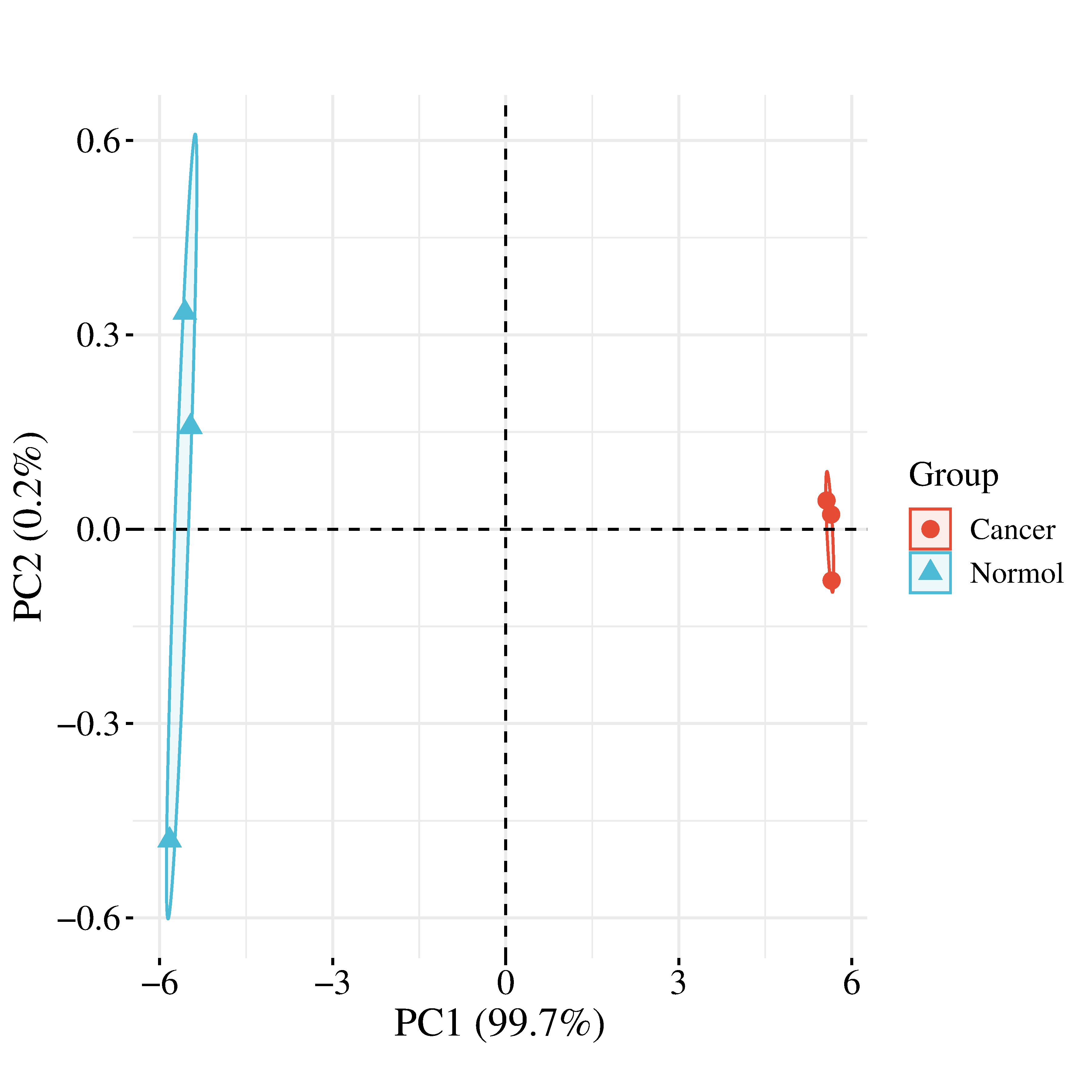

Supplement: Supplementary file 1 [file Image1.tiff]

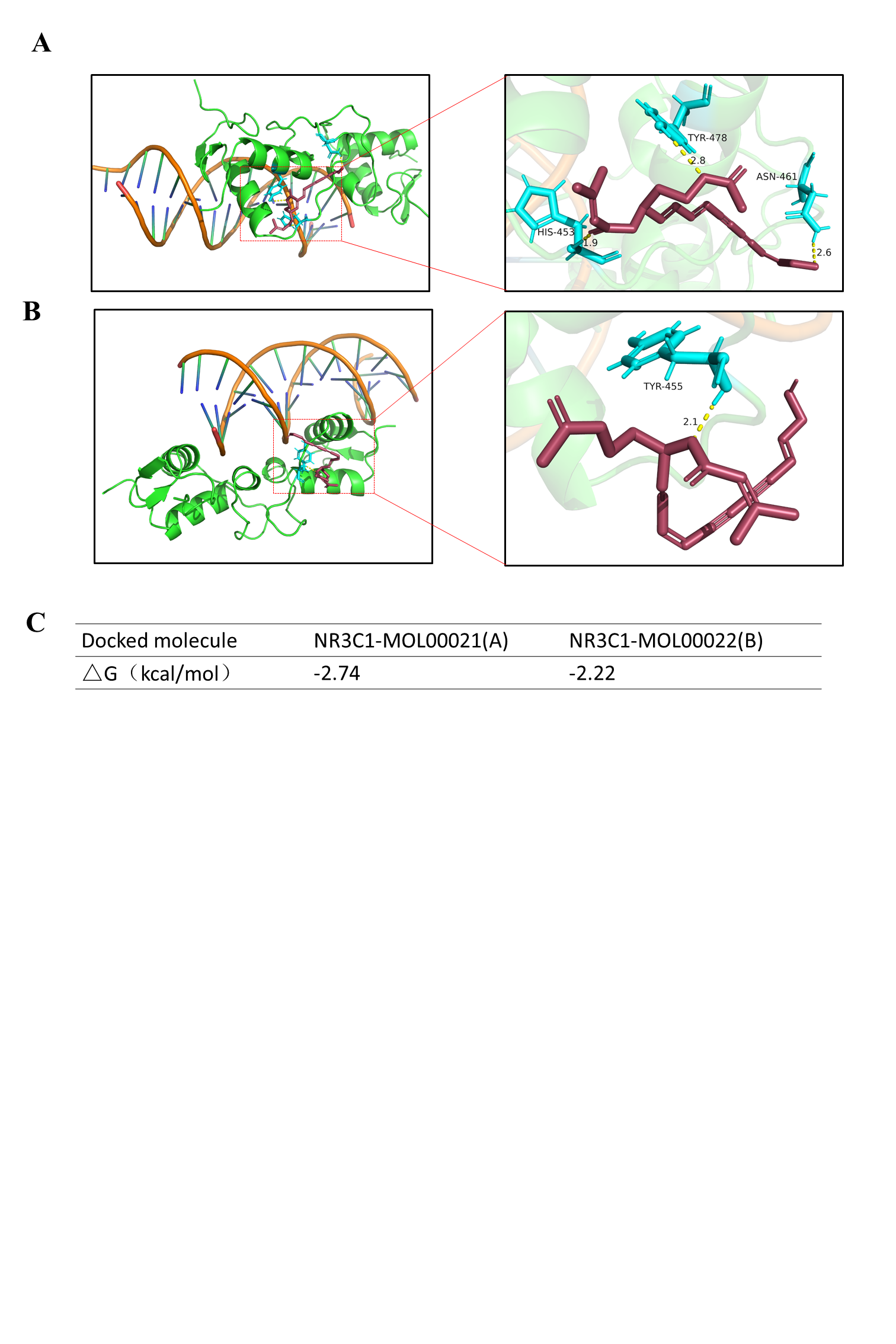

Supplement: Supplementary file 2 [file Image3.tif]

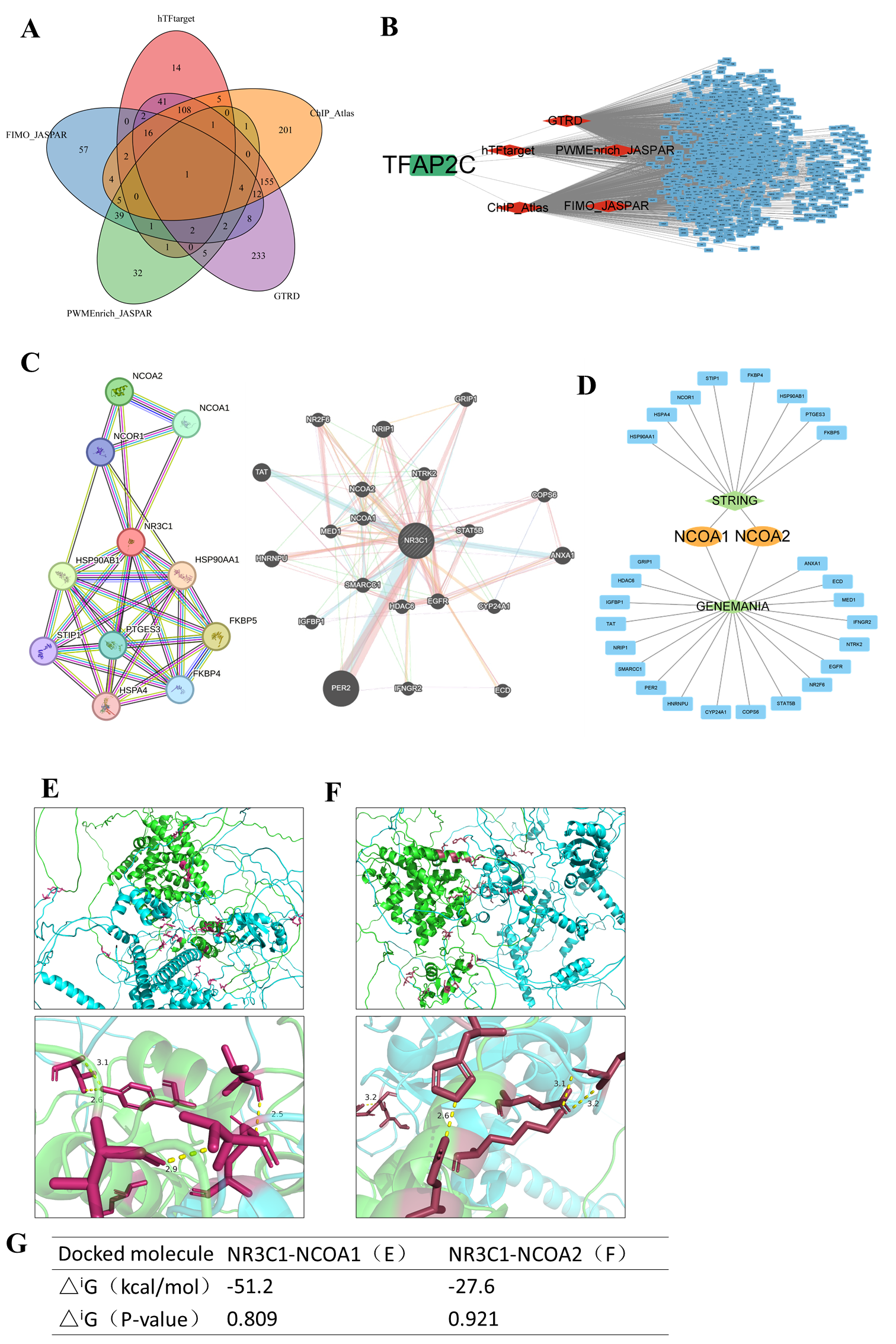

Supplement: Supplementary file 3 [file Image4.tif]

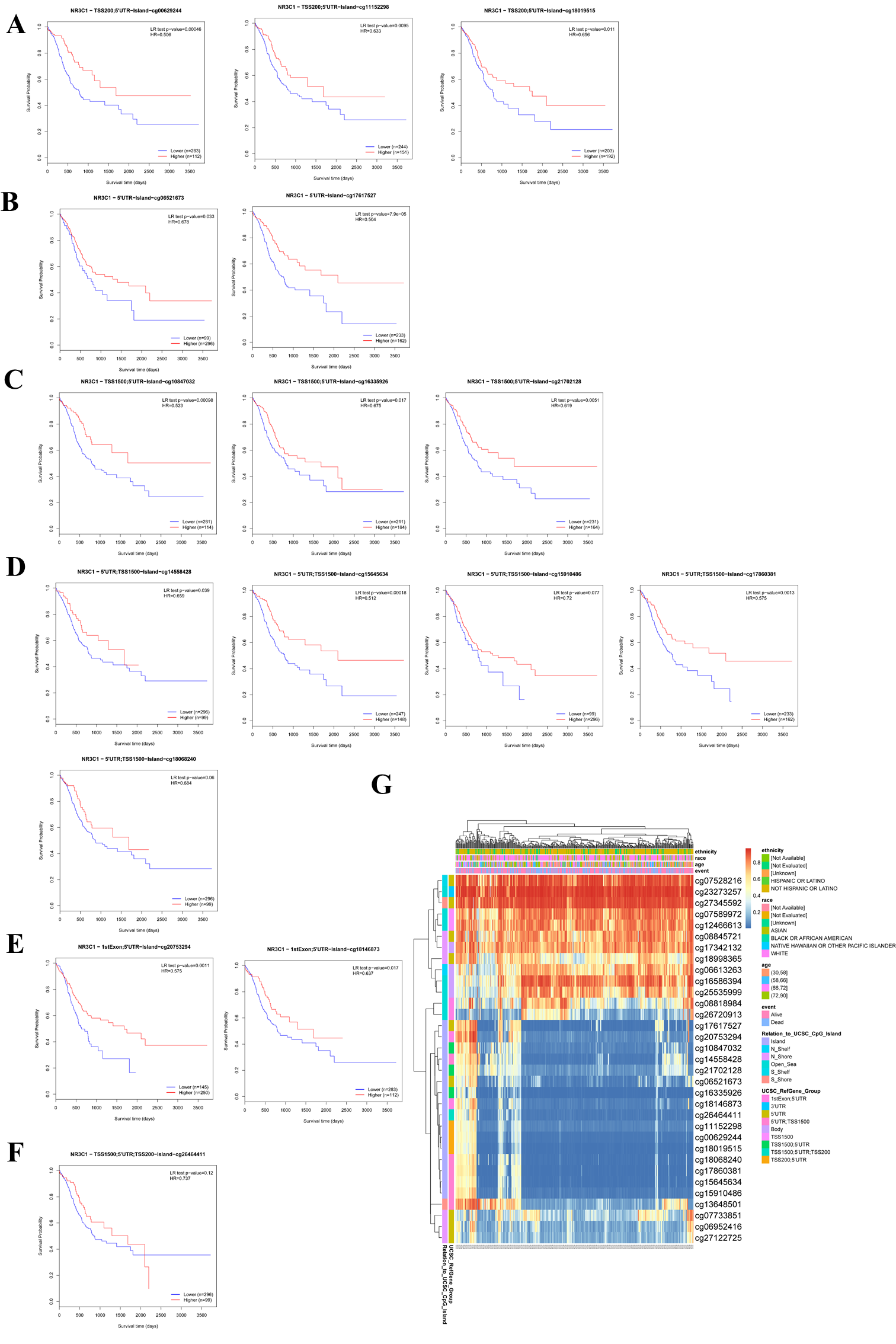

Supplement: Supplementary file 4 [file Image2.tif]
